# Differentiating commensal and disease-associated *Enterococcus cecorum* isolates in poultry using protein sequences

**DOI:** 10.3389/fvets.2026.1850860

**Published:** 2026-07-09

**Authors:** Moses B. Ayoola, B. Santhana Krishnan, Bindu Nanduri, Douglas D. Rhoads, Mahalingam Ramkumar

**Affiliations:** 1 Department of Comparative Biomedical Sciences, College of Veterinary Medicine, Mississippi State University, Starkville, MS, United States; 2 Department of Biological Sciences, University of Arkansas, Fayetteville, AR, United States; 3 Department of Computer Science and Engineering, Mississippi State University, Starkville, MS, United States

**Keywords:** amino acid k-mers, bacterial chondronecrosis with osteomyelitis, *Enterococcus cecorum*, k-mer analysis, poultry infection, sepsis, supervised learning algorithms

## Abstract

*Enterococcus cecorum* (EC) is an avian enteric commensal, but around the year 2000, it emerged as a frequent causative agent of bacterial chondronecrosis with osteomyelitis (BCO) in older broilers. More recently, around 2020, EC has also been isolated from cases of sepsis (SS) in young birds. Differentiating pathogenic from commensal isolates has therefore become critical for surveillance and control efforts. In this study, we applied amino acid k-mer profiling (*k* = 5) along with supervised learning algorithms, including Random Forest and Multilayer Perceptron approaches, to identify minimal sets of discriminatory oligopeptides associated with pathogenicity. Using only five k-mers, we distinguished commensal isolates (NONE, *n* = 80) from all pathogenic isolates (BCO + SS, *n* = 146) with 86% accuracy. In addition, comparisons between BCO (*n* = 113) and SS (*n* = 33) isolates were classified with 90% accuracy using eight k-mers. The discriminatory k-mers mapped to proteins involved in carbohydrate transport, stress response, mobile genetic elements, and metabolic remodeling, reflecting niche-specific evolutionary pressures. Sepsis isolates showed signatures associated with acute systemic pathogenicity, while BCO isolates were enriched for proteins that support chronic persistence in bone tissue. These findings provide new insights into *E. cecorum* pathogenesis and highlight the potential utility of supervised learning approaches in resolving subtle but functionally relevant proteomic differences. The results have implications for improving targeted interventions and monitoring strategies in poultry production.

## 1 Introduction

*Enterococcus cecorum* (EC) is a Gram-positive bacterium and intestinal commensal known to cause skeletal disease (bacterial chondronecrosis with osteomyelitis, BCO) in older broiler chickens (1, 2). However, pathogenic EC isolates have recently emerged, causing septicemia and mortality in younger broiler flocks (3–5). Collectively, EC infections are associated with significant economic losses due to reduced performance, increased mortality, and carcass condemnations (6). EC-associated disease has increasingly been reported in commercial broiler systems across multiple countries, including Europe and North America, and is now recognized as an emerging poultry health concern. Affected flocks may experience cumulative mortality rates of 5–15%, with 25–35% of birds affected during outbreaks (7). EC disease progresses in two phases: an initial septic phase during the first 3 weeks, often marked by subclinical systemic infection, followed by a skeletal phase from week 3 onward, characterized by lameness, paralysis, and osteomyelitis (8). Gut commensalism of EC generally initiates at approximately 3 weeks of age (9). This represents the classical timing of intestinal commensal establishment during normal gut maturation. Compared with commensals, early gut colonization by pathogenic isolates gives them a competitive advantage, possibly enabling systemic spread (8, 10). Pathogenic EC has been found in organs (gut, spleen, and yolk sac) as early as 3 days of age (8, 10). This early detection reflects systemic dissemination prior to or independent of typical commensal gut colonization. Concurrent gut damage may facilitate EC translocation into the bloodstream and bone (11). EC has also been occasionally isolated from mammals and reported in sporadic opportunistic infections in humans, although its zoonotic significance and transmission routes remain unclear (12, 13). Pathogenic EC isolates show greater resistance to environmental stressors and host defenses, which may aid survival during embryogenesis (14). Discontinuation of growth-promoting antibiotics in poultry production may have contributed to increased EC disease, and no vaccines are currently available for EC in poultry.

Despite multiple approaches for differentiating commensal from pathogenic EC, no single method has consistently distinguished these groups across studies. Existing approaches include embryo lethality assays (7, 15), pulsed-field gel electrophoresis (1), comparative genomics (16), and metabolic profiling (17). However, findings from these approaches are often inconsistent. For example, some isolates classified as pathogenic using embryo lethality assays do not cause disease in live birds (7). Similarly, genomic and metabolic differences between isolates do not consistently predict disease association (16, 17). Additional complexity arises from phenotypic variability, including the emergence of small colony variants with altered disease-associated behavior (17). These observations highlight the need for more integrative and standardized approaches to isolate classification. *Enterococcus* genomes are highly plastic and frequently undergo horizontal gene transfer, acquisition of mobile genetic elements, and genomic rearrangements. Such genomic flexibility complicates the identification of stable or universal markers associated with disease phenotypes. Consequently, relationships between genomic features and disease outcomes are unlikely to be strictly deterministic. However, this variation is not entirely random and may still produce reproducible patterns that can support classification. Importantly, the distinction between commensal and pathogenic EC is not absolute. Individual isolates may behave as gut commensals under some conditions while retaining the potential to contribute to disease under specific host or environmental circumstances.

In our previous study, phylogenomic and pangenomic analyses of 227 EC isolates revealed that sepsis (SS) isolates have diverse evolutionary ancestors but are still closely related to both commensal (NONE; both terms are used interchangeably henceforth) and BCO isolates, with no unique gene acquisitions consistently distinguishing SS isolates (3). However, single-nucleotide polymorphism (SNP) analyses identified missense mutations in protein-coding genes, potentially affecting stress response and immune evasion, which were enriched in SS isolates, supporting a mutational basis for pathogenicity. Although a previously described 12-gene capsular cluster was common in BCO and SS isolates, it was not universally present and is not sufficient for diagnosis (3, 18). A key limitation of the previous study was its reliance on gene presence/absence patterns and core genome SNP-based phylogenetics, which, while informative, may not fully capture the subtle or distributed genomic signals that distinguish pathogenic from commensal EC isolates. The polyphyletic nature of pathogenic isolates and the lack of universally present pathogenicity genes or gene clusters among SS and BCO isolates suggest that conventional approaches may overlook functionally relevant variation. To overcome these constraints, we used an alternative approach leveraging amino acid k-mer profiling combined with supervised learning approaches to identify minimal sets of proteomic features that can distinguish between commensal and pathogenic (BCO + SS) isolates, and further distinguish between BCO and SS pathotypes. This method has the potential to reveal non-obvious, protein-level variations and complex patterns of pathogenicity signatures in the proteomes, ultimately advancing our ability to classify isolates, predict pathogenic potential, and inform more targeted strategies for disease mitigation.

## 2 Methods

### 2.1 Dataset

At the time of analysis (August 2025), 227 *E. cecorum* proteomes were available at NCBI. Of these, 80 proteomes were classified as NONE (intestinal isolate or no disease association known), 33 as SS, and 113 as BCO. One proteome annotated as both SS and BCO was excluded to maintain mutually exclusive class labels, although its existence highlights the potential overlap between disease phenotypes. After exclusion, a total of 226 proteomes were used for analysis. Proteomes were generated from *de novo* genome assemblies annotated with Prokka (v1.15.6, (19)) and exported in FASTA format (3). Proteins annotated as hypothetical were further investigated using NCBI BLASTP (20), UniProt (21), and Bakta (v1.12.0, (22)) to identify potential functional annotations. Proteins annotated as hypothetical were further confirmed using InterProScan (23) and AlphaFold (24) analysis. Metadata curation involved cross-referencing NCBI BioSample annotations, removal of inconsistent or ambiguous disease labels, and harmonization of isolate classification based on the published literature when available. Only isolates with clearly defined phenotypic categories (NONE, SS, and BCO) were retained.

### 2.2 K-mer feature generation and filtering

**Figure 1:**
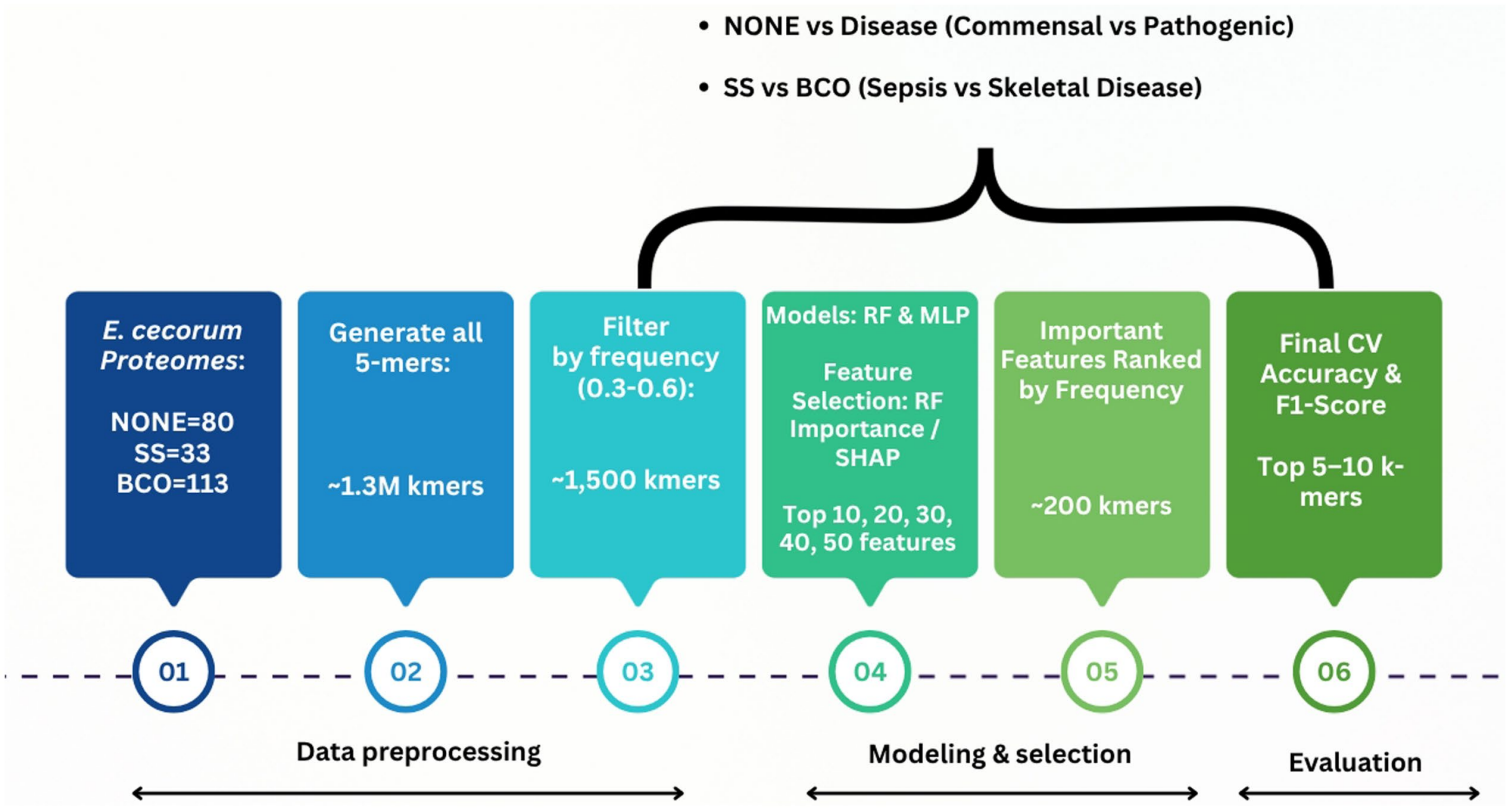
Workflow for data processing, feature selection, and model evaluation. Proteomes from *E. cecorum* isolates (NONE = 80, SS = 33, BCO = 113) were processed to generate all possible 5-mers (~1.3 million). After filtering by frequency distribution thresholds (0.3–0.6), ~1,500 informative 5-mers were retained. Random forest (RF) and multi-layer perceptron (MLP) models were applied with feature selection using RF importance and SHAP values, evaluating subsets of the top 10–50 features. Selected features were combined and ranked by frequency of selection. Final cross-validation accuracy and F1-scores were computed using the top 5–10 most frequently selected k-mers.

To investigate protein-level variation, we generated all possible amino acid k-mers (*k* = 5) from the proteomes of each isolate, producing a binary presence/absence matrix in which each column represented a unique k-mer and each row represented a proteome. This k-mer length was selected as an optimal balance between biological relevance and computational feasibility. Five-residue peptides are long enough to capture informative motifs, such as partial functional domains, binding interfaces, or membrane-associated signals, and short enough to allow exhaustive, non-redundant enumeration across protein-coding regions. Larger k values (e.g., k = 6–7) greatly increase the theoretical feature space (64 million to 1.28 billion possible k-mers) and introduce sparsity and overfitting risk compared with the dataset size. We selected amino acid sequences rather than nucleotide sequences because they provide a more direct link to protein structure and function. Nucleotide k-mers can be confounded by synonymous substitutions that do not alter protein function, whereas amino acid k-mers more accurately reflect functional variation relevant to phenotype. Previous studies have shown the effectiveness of short amino acid k-mers in microbial proteomics and machine learning applications. For example, ValizadehAslani et al. (25) directly evaluated the impact of amino acid k-mer length on classification performance and reported that “amino acid 5-mers always get the best performance” in their comparative proteomic analyses. This empirical support reinforces our choice of 5-mers as informative and computationally tractable features for distinguishing phenotypic subtypes of EC. Isolates were grouped into disease-associated isolates (SS and BCO) and non-disease isolates (NONE). For each k-mer, we calculated its frequency of presence within both the Disease and NONE groups. To reduce noise and focus on potentially informative features, k-mers were retained based on the absolute difference in frequency between the groups. This step served as an initial unsupervised filtering procedure applied prior to model training. Several threshold values (0.3–0.6) were evaluated in a sensitivity analysis framework to assess the robustness of downstream model performance, with no intent to optimize performance on a specific test set. Figure 1 summarizes the overall analysis pipeline.

### 2.3 Feature selection and model evaluation

A value of 1 indicates the presence of k-mer in at least one protein within the isolate, whereas 0 indicates its absence entirely. These features were treated strictly as unweighted, unnormalized binary indicators of k-mer occurrence.

This matrix was used as the feature input for supervised learning under two classification schemes:

- NONE (0) vs. Disease (BCO + SS, 1), which distinguishes commensal isolates from all pathogenic isolates and.
- SS (0) vs. BCO (1), which distinguishes systemic infection from skeletal disease isolates.

Two classification models were used: a Random Forest (RF) with 100–200 estimators and a Multi-Layer Perceptron (MLP) with two hidden layers of 100 and 50 neurons. Feature selection was performed within each fold using either RF feature importance or SHapley Additive exPlanations (SHAP) values (26, 27). For each model and fold, k-mers (candidate numbers tested: 10, 20, 30, 40, and 50) were ranked based on importance scores derived exclusively from the training data. These selected features were then used to train the model on the training fold, and performance was assessed on the held-out test fold using a weighted F1-score. Across folds, the frequency of selection of each k-mer was recorded to estimate feature stability across cross-validation iterations. This aggregation was performed after cross-validation and did not involve any test-fold information in the feature ranking. Subsequently, an independent final cross-validation analysis was conducted using the top 5–10 most frequently selected k-mers identified from the training-fold rankings. The resulting cross-validated accuracy and F1-scores were used to identify the minimal feature set yielding optimal classification performance. The best-performing feature configuration, along with its mean accuracy and F1-score, was reported for downstream analyses. Model performance was evaluated using nested cross-validation; however, no fully independent external validation dataset was available. Thus, the reported performance reflects internal validation within the current dataset.

### 2.4 Annotation of identified important k-mers

The discriminatory k-mers identified through feature selection were mapped to protein sequences using a custom Python script implemented with Biopython (v1.87). Protein sequences were parsed from the corresponding FASTA (.faa) files using the SeqIO module. For each k-mer, exact substring matching was performed against all protein sequences using Python’s native string search function. When a match was identified, the corresponding protein annotation was retrieved from the FASTA header to provide functional context. In addition, the genomic location of each k-mer within the protein sequence was recorded, along with a ± 10 amino acid flanking region to capture local sequence context. The output results were exported as tab-delimited CSV files for downstream analysis. No external alignment tool was used for k-mer mapping, as the analysis relied on exact sequence matching within translated protein sequences.

### 2.5 Extraction of specific polypeptides containing k-mers

Predicted proteomes in FASTA (.faa) format were grouped based on disease association. Protein sequences corresponding to specific k-mers or annotated motifs were extracted using a custom Bash pipeline (extract_faa.sh; Supplementary Data) implemented with seqkit v2.3.0 (28). FASTA files were converted to tab-delimited format using seqkit fx2tab, and matching sequences were retrieved using case-insensitive pattern searching with grep. Extracted sequences were converted back to FASTA format using seqkit tab2fx and manually inspected for consistency. Sequence headers were standardized to include trait labels prior to downstream analysis. Multiple sequence alignments of extracted protein sets were performed using Clustal Omega v1.2.4 (29) with default protein parameters and full distance matrix computation enabled. The resulting alignments were exported in MSF format. Phylogenetic trees were inferred from the multiple sequence alignments using IQ-TREE v3.0.1 (30). Model selection was performed automatically using ModelFinder (MFP option), and branch support was assessed using 1,000 ultrafast bootstrap replicates. Trees were inferred under the best-fit amino acid substitution model selected by IQ-TREE, with computation performed under default optimization settings and automatic thread allocation. The script extract_faa.sh was developed using ChatGPT (GPT-5.1) as an assistive tool for code drafting and optimization and was subsequently reviewed, validated, and manually verified by the authors prior to execution and use in the analysis.

## 3 Results

### 3.1 Models to discriminate commensal from pathogenic isolates

#### 3.1.1 Model performance and feature selection in commensal vs. pathogenic isolates

**Figure 2:**
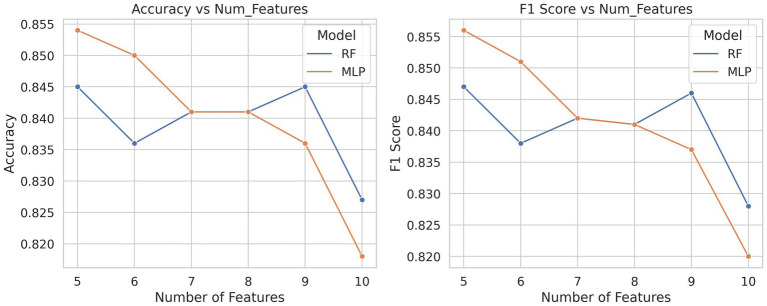
Model performance and feature selection for discrimination of commensal vs. pathogenic *E. cecorum* isolates. Line plots show the accuracy (left) and F1-score (right) of random forest (RF) and multi-layer perceptron (MLP) models as a function of the number of k-mer features used. Both classifiers achieved their highest performance when restricted to only five features, with MLP reaching an average accuracy and F1-score of ~86%.

Our model evaluation demonstrated that accurate discrimination between commensal and pathogenic EC isolates can be achieved with a relatively small number of k-mers. As shown in Figure 2, the MLP classifier achieved the best overall performance when trained with just five k-mers, yielding a mean accuracy and F1-score of approximately 86% based on cross-validation performance across folds. RF models also performed robustly with five k-mers, although their peak performance was slightly lower than that of the MLP model. We observed that the performance of both classifiers declined once the feature set exceeded five k-mers.

#### 3.1.2 Discriminative k-mers in commensal vs. pathogenic isolates

To further explore how individual k-mers contributed to classification, we examined their distribution across commensal and pathogenic isolates (Table 1; Supplementary Table S1). While four k-mers showed clear enrichment in pathogenic proteomes, the LDATV k-mer was more prevalent in commensal isolates. For instance, SFDLF and FTNFE were detected in approximately 87% of pathogenic isolates but occurred in only 29–36% of commensals. In contrast, LDATV was found in 75% of commensal proteomes and only 15% of pathogenic proteomes.

**Table 1:** Frequencies of top discriminative k-mers in commensal vs. pathogenic proteomes.

K-mer	Count in disease (*n* = 146)	Count in NONE (*n* = 80)	Percentage in disease	Percentage in none
SFDLF	127	23	86.99	28.75
KNYKK	122	17	83.56	21.25
LDATV	22	60	15.07	75.00
ENIHL	116	9	79.45	11.25
FTNFE	127	29	86.99	36.25

#### 3.1.3 Protein mapping of discriminatory k-mers in commensal vs. pathogenic isolates

**Figure 3:**
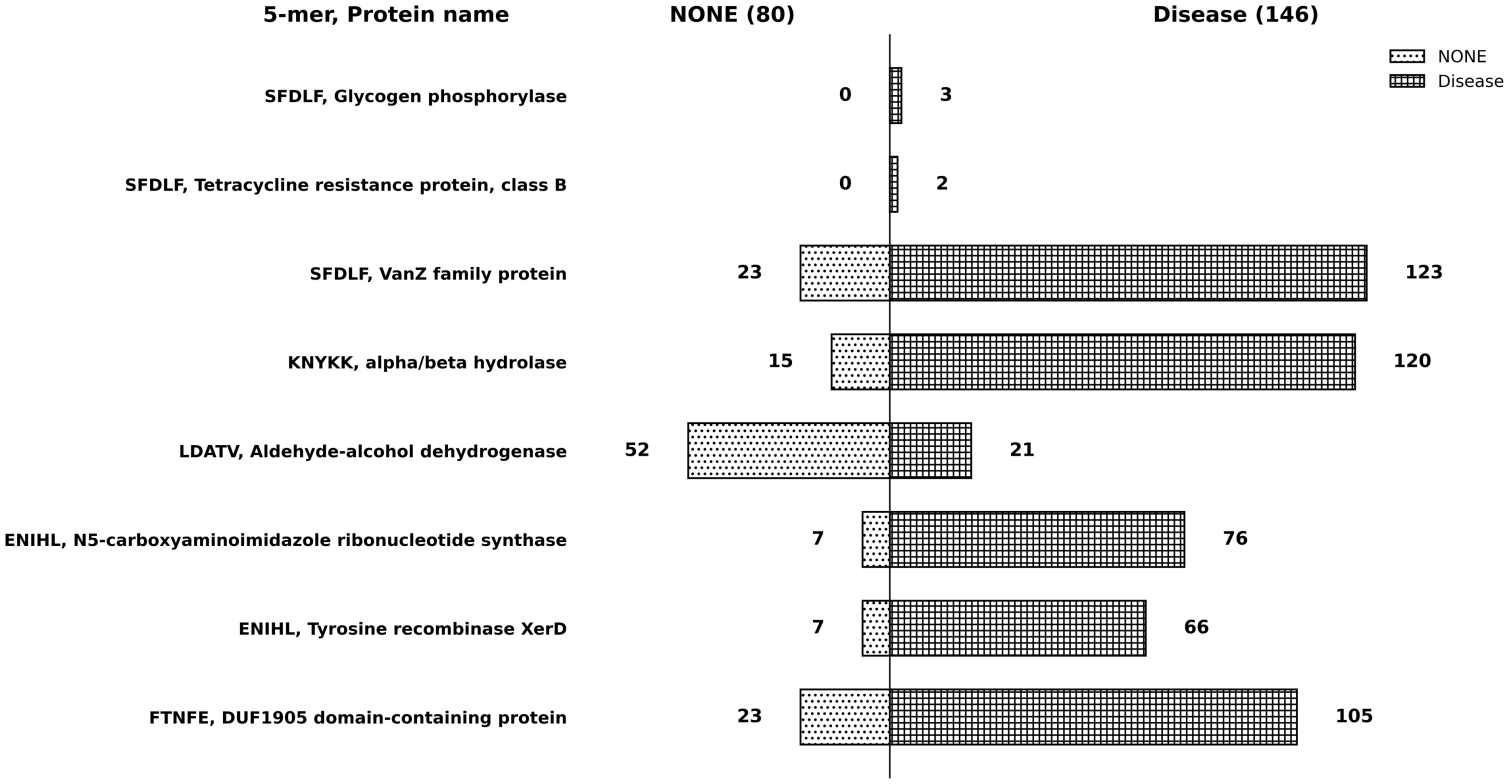
Functional annotation and distribution of top discriminatory k-mers in commensal (dotted) vs. pathogenic (checkered) *E. cecorum* proteomes. Each row represents a distinct k-mer mapped to a protein (with a specific length, k-mer starting position, and amino-terminal dodecapeptide recognized by its paralogs), and the corresponding bars indicate the number of proteomes in each group containing that k-mer. The k-mers are associated with proteins involved in antimicrobial resistance, metabolism, genome maintenance, and genome plasticity, highlighting potential functional signatures that distinguish pathogenic from commensal isolates.

The discriminatory k-mers identified in the NONE vs. Disease comparison mapped to several annotated proteins (Figure 3). Specifically, LDATV mapped to aldehyde-alcohol dehydrogenase; SFDLF mapped to glycogen phosphorylase, tetracycline resistance protein, and VanZ family protein; ENIHL mapped to N5-carboxyaminoimidazole ribonucleotide synthase and tyrosine recombinase XerD; whereas KNYKK and FTNFE mapped to alpha/beta hydrolase and DUF1905 domain-containing proteins, respectively.

### 3.2 Models to discriminate SS from BCO isolates

#### 3.2.1 Model performance and feature selection in SS vs. BCO isolates

**Figure 4:**
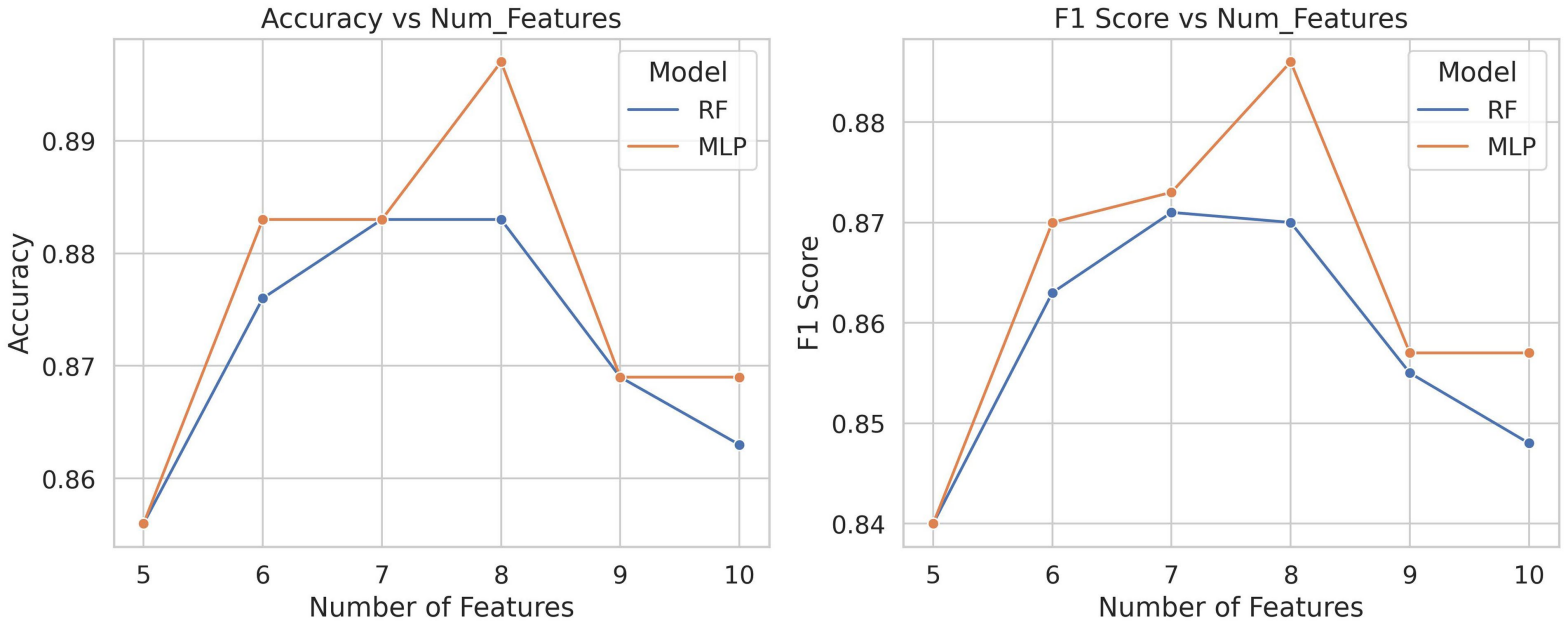
Model performance and feature selection for discrimination of SS vs. BCO *E. cecorum* isolates. Line plots show the accuracy (left) and F1-score (right) of Random Forest (RF) and multi-layer perceptron (MLP) models as a function of the number of k-mer features used. Optimal performance was achieved with 7–8 features for both models, indicating strong discriminatory power between isolates associated with bacterial chondronecrosis with osteomyelitis (BCO) and sepsis (SS). Using eight features, the MLP model achieved approximately 90% accuracy, outperforming the RF model.

As shown in Figure 4, both RF and MLP models achieved optimal accuracy and F1-score using seven to eight k-mers. The MLP model outperformed RF, achieving ~90% accuracy with eight k-mers. Performance for both models declined when more than eight k-mers were included.

#### 3.2.2 Discriminative k-mers in SS vs. BCO isolates

Table 2 summarizes the counts and percentage occurrence of eight k-mers across BCO and SS EC proteomes (Supplementary Table S2). Five k-mers (ILLAF, KVNSR, NFTCL, LEEKK, and AAKRT) are highly enriched in BCO isolates, appearing in 82–93% of samples, whereas three k-mers (REIDK, DSGTI, and RKEKI) are more frequent in SS isolates, with occurrence rates of 51–76%.

**Table 2:** Frequency of top discriminatory k-mers in BCO vs. SS *E. cecorum* proteomes.

K-mer	Count in ^*^BCO (*n* = 113)	Count in ^+^SS (*n* = 33)	Percentage in ^*^BCO	Percentage in ^+^SS
ILLAF	100	15	88.50	45.45
KVNSR	93	12	82.30	36.36
NFTCL	99	15	87.61	45.45
REIDK	9	17	7.96	51.52
DSGTI	14	18	12.39	54.55
LEEKK	99	14	87.61	42.42
RKEKI	22	25	19.47	75.76
AAKRT	105	16	92.92	48.48

*BCO: bacterial chondronecrosis with osteomyelitis; +SS: Sepsis.

#### 3.2.3 Protein mapping of discriminatory k-mers in SS vs. BCO isolates

**Figure 5:**
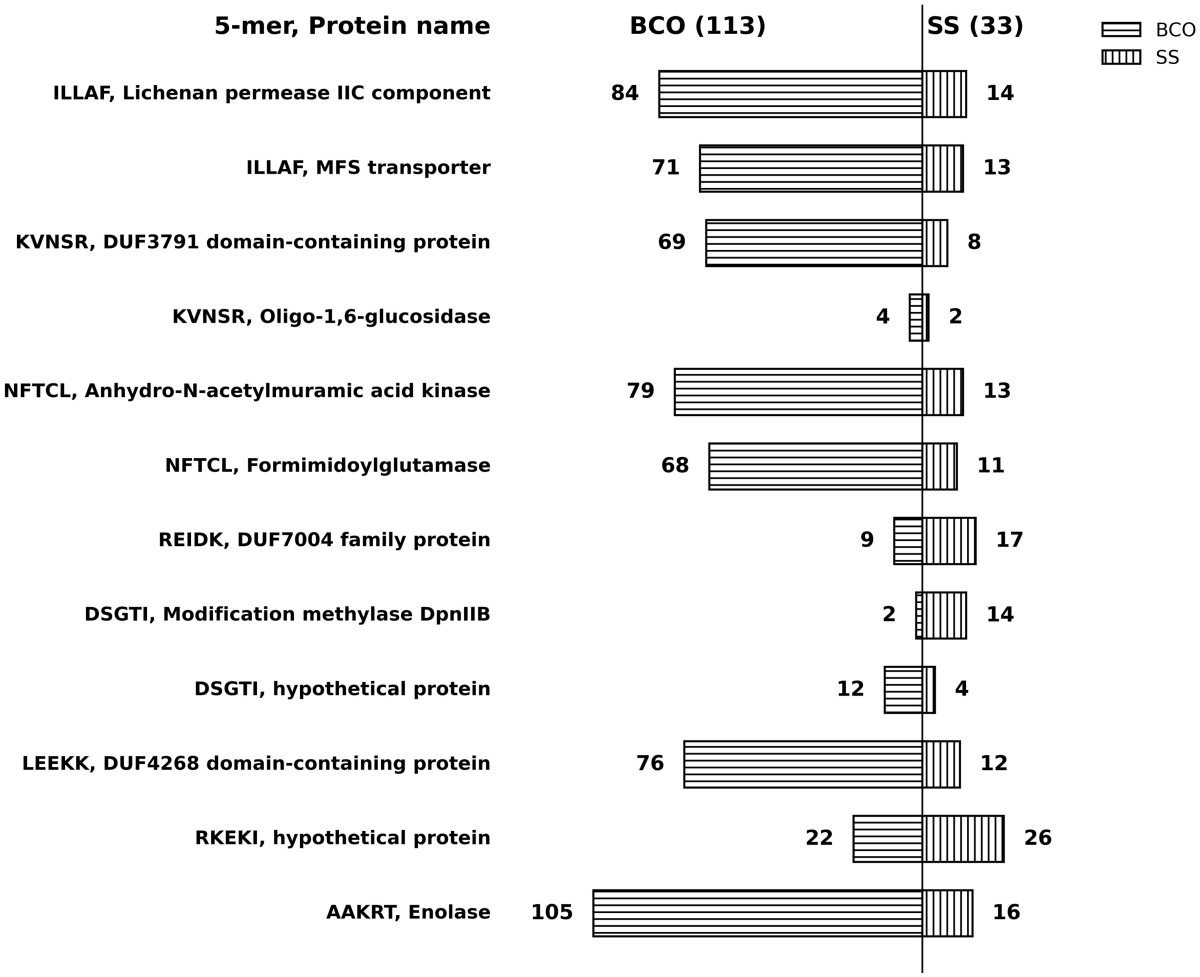
Functional annotation and distribution of top discriminatory k-mers in BCO (horizontal lines) and sepsis (SS, vertical lines) *E. cecorum* proteomes. Each row represents a distinct k-mer mapped to a protein (with a specific length, k-mer starting position, and amino-terminal dodecapeptide to recognize paralogs), and the bars indicate the number of proteomes in each group containing that k-mer. BCO-enriched k-mers are associated with transporters and metabolic enzymes, whereas SS-enriched k-mers are associated with immune evasion, genome defense, or horizontal gene transfer, reflecting the distinct pathogenic strategies between the two disease phenotypes.

K-mers enriched in BCO and SS isolates are mapped to several annotated proteins (Figure 5). BCO-associated k-mers mapped to lichenan permease IIC component, oligo-1,6-glucosidase, major facilitator superfamily transporter, formimidoylglutamase, enolase, anhydro-N-acetylmuramic acid kinase, and DUF-containing proteins. SS-associated k-mers mapped primarily to a DNA modification methylase (DpnIIB).

### 3.3 Investigation of the specific basis of discriminatory k-mers

To better understand the biological basis of discriminatory k-mers and determine whether they reflect gene presence/absence, paralog-specific distribution, or sequence variation within orthologous proteins, we performed a targeted follow-up analysis of five proteins that showed the most significant differences. For NONE vs. Disease, we selected VanZ family protein (VanZ; SFDLF), alpha/beta hydrolase (Abh; KNYKK), and DUF1905 domain-containing protein (Duf1905; FTNFE). For BCO vs. SS, these were lichenan permease IIC component (LicC3; ILLAF) and anhydro-N-acetylmuramic acid kinase (AnmK; NFTCL). Protein sequences were extracted based on annotation names or sequence motifs. The predicted polypeptide sequences were compared for length, starting position of k-mer, and amino-terminal dodecapeptide to distinguish between paralogs and assess k-mer presence or absence.

For VanZ in NONE vs. Disease, extraction of all polypeptides containing SFDLF identified 145 of 226 genomes that contained a 200-residue ortholog, with the SFDLF from positions101 to 105. All orthologs contained a FALFF k-mer, so we extracted all polypeptides containing that k-mer with 190 representatives of the VanZ ortholog (78/80 NONE; 112/146 Disease). Orthologs that lacked SFDLF at positions 101 to 105 contained either SYDLF or SFELF. The alternative SFELF with FALFF at positions 5–9 was present in 62% (50/80) of the NONE genomes vs. 16% (23/146) of the Disease genomes. The alternative SYDLF with FALFF at positions 5 to 9 was present in 6.2% (5/80) of the NONE genomes only. VanZ orthologs were recovered from 112 of 145 genomes, but only the SFDLF-containing isoform. Thus, the SFDLF-containing ortholog was preferentially found in the Disease isolate genomes (84%; 122/146) compared with the NONE genomes (29%; 23/80).

For Abh in NONE vs. Disease, the extraction of all polypeptides containing KNYKK identified four different polypeptides based on length and amino terminus. This identified one polypeptide, which we labeled Abh1, comprising 284 residues with the KNYKK motif from residues 2 to 6. The Abh1 polypeptide was present in 19% of NONE genomes (15/80), while it was present in 82% of Disease genomes (120/146). To further investigate, we identified a KVTGK motif at position 92, present in all Abh1 orthologs, and used it to extract 1,042 polypeptides containing this k-mer, providing 219 orthologs of Abh1 (of 226 total genomes), of which 84 orthologs contained the alternatives TNYKK or INYKK in place of the KNYKK from residues 2 to 6. Analysis by trait groups showed that 19% of NONE genomes (15/80) contained the KNYKK motif, while 82% of Disease genomes (120/146) contained the KNYKK motif. Conversely, 73% of NONE genomes (58/80) contained the TNYKK version of Abh1, compared with only 16% of Disease genomes (23/146). The INYKK versions of Abh1 were less numerous, representing 3% of NONE genomes and 1% of Disease genomes.

For DUF1905 in NONE vs. Disease, extraction based on the FTNFE motif identified 163 polypeptides with one paralog of 110 residues from 129 genomes. The amino-terminal MDSIQD hexapeptide was used to extract 192 polypeptides containing this sequence, all being similar in length (range: 91–120) and sequence. The 129 Disease genomes had a predicted protein of 110 residues, while ortholog length variation was limited to 9 of 63 orthologs from the NONE genomes. Length polymorphisms were limited to variations in the carboxy-terminal sequences. The FTNFE motif at positions 7–11 had one alternative, FTNYT. Orthologs for this polypeptide were equally recovered from both traits (63/80 = 79% NONE; 128/146 = 88% Disease). The FTNFE-containing ortholog was more prevalent in Disease (72%; 105/146) vs. NONE (29%; 23/80). The FTNYT ortholog was more prevalent in NONE (50%; 40/80) vs. Disease (16%; 23/146).

For LicC, there were seven different paralogs based on length and amino terminus, with isolate genomes containing between 1 and 6 paralogs. However, only paralog 3 (LicC3; 431 amino acids) contained ILLAF at positions 302–306. There were no instances of LicC3 lacking the ILLAF k-mer. LicC3 was present in 74% of BCO genomes (84/113) and 42% of SS genomes (14/33). Thus, the ILLAF k-mer was diagnostic for the presence or absence of this paralog and not due to sequence polymorphisms within this paralog. For AnmK in BCO vs. SS, there was only one ortholog of 380 amino acids. AnmK was differentially represented in 75% of BCO (85/113) vs. 42% of SS genomes (14/33). However, the NFTCL k-mer (residues 34–38) was present in only 93% (79/85 in BCO and 13/14 in SS) of the orthologs irrespective of disease phenotype. Considering only the NFTCL-containing AnmK, the prevalence was 70% in BCO genomes (79/113) vs. 39% in SS (13/33).

## 4 Discussion

The above findings highlight that a limited set of protein-level k-mers can differentiate EC isolate groups with moderate-to-high classification performance within the analyzed dataset. Although the biological roles of these discriminatory k-mers remain to be experimentally validated, which is beyond the scope of the present study, their repeated selection across cross-validation folds provides confidence that they represent reproducible statistical signatures. While all identified discriminative k-mers were observed in both isolate classes, their differing frequencies suggest distinct enrichment patterns between pathogenic and commensal isolates. Since the classification framework relies on the combined presence patterns of multiple k-mers rather than the discriminatory capacity of any single feature, these k-mers should not be interpreted as standalone diagnostic markers, but rather as components of a multivariate signature that collectively improve group separation. Mapping of the most discriminative k-mers to proteomic features revealed associations with proteins involved in antimicrobial resistance, carbohydrate metabolism, and genetic recombination (Figure 3). The association between LDATV and aldehyde-alcohol dehydrogenase may reflect enhanced metabolic activity in commensal EC, potentially contributing to gut homeostasis (31). SFDLF mapping to metabolic enzymes such as glycogen phosphorylase suggests that pathogenic isolates may exploit expanded metabolic flexibility during colonization and infection. Its mapping to tetracycline resistance protein (32) and vanZ family protein (33) is consistent with the role of antimicrobial resistance in pathogen adaptation and persistence under selective pressures that may enhance survival in host-associated environments. The enrichment of ENIHL-associated proteins involved in nucleotide biosynthesis and recombination may indicate enhanced metabolic flexibility and genome adaptation in pathogenic isolates (34). K-mer ENIHL also mapped to tyrosine recombinase XerD, which was enriched in the Disease group and is known to mediate genome rearrangements, horizontal gene transfer, and the acquisition of mobile genetic elements (35). The enrichment of SFDLF and ENIHL in pathogenic isolates supports the hypothesis that pathogenic EC isolates maintain greater genome plasticity, facilitating adaptation to host pressures and enhancing pathogenicity potential. KNYKK mapped to alpha/beta hydrolases that are versatile enzymes involved in lipid metabolism, membrane remodeling, immune modulation, and stress adaptation in other bacteria (36, 37). For example, in *Mycobacterium tuberculosis*, alpha/beta hydrolases participate in cell envelope maintenance and extracellular lipase activity (38), while in Gram-negative pathogens, they can act as pathogenicity factors that modify host membranes or immune responses (39). Based on these precedents, the alpha/beta hydrolases identified in this study could potentially contribute to EC pathogenicity through similar mechanisms. The association between FTNFE and a DUF1905 domain-containing protein suggests a potentially uncharacterized function associated with pathogenic isolates, although experimental validation is needed. These candidates require further experimental investigation, including mutagenesis, biochemical characterization, and host response studies.

K-mers highly enriched in BCO proteomes (Figure 5) mapped to a lichenan permease IIC component, likely part of a carbohydrate-specific phosphotransferase system (PTS) (40), and to oligo-1,6-glucosidase, an enzyme that hydrolyzes *α*-1,6 linkages in oligosaccharides (41). These functions suggest an expanded capacity for complex carbohydrate import and degradation, potentially enhancing bacterial fitness in the nutrient-limited, inflamed microenvironment of necrotic bone lesions. The use of host-derived glycans or structurally complex environmental carbohydrates may be a key survival strategy in BCO pathogenesis. Furthermore, BCO-associated isolates were enriched for a broader set of proteins with functions supporting persistence in nutrient-limited, inflamed bone tissue. These included a major facilitator superfamily (MFS) transporter, likely involved in nutrient uptake or antimicrobial efflux (42), and formimidoylglutamase, a key enzyme in histidine degradation (43), which may provide metabolic flexibility in specialized tissue niches. Additionally, k-mers mapping to anhydro-N-acetylmuramic acid kinase, a component of the peptidoglycan recycling pathway, suggest enhanced cell wall remodeling or survival during host-induced stress (44). Enolase, enriched in BCO isolates, may serve both its canonical role in glycolysis and a non-canonical function in host interaction, such as plasminogen binding and tissue adhesion (45, 46). Finally, DUF3791 and DUF4268 domain-containing proteins were also more prevalent in BCO proteomes. While their exact function is unknown, their association with bone-pathogenic isolates suggests a potential role in host adaptation or niche-specific fitness, warranting further study. In contrast, SS-associated isolates were enriched for k-mers mapping to a DNA modification methylase (DpnIIB), a DNA methyltransferase involved in restriction-modification systems. This protein may facilitate immune evasion, genome defense, or horizontal gene transfer (47), potentially enhancing adaptability and systemic invasion in young, immunologically immature broilers. Together, these findings underscore the functional divergence between SS- and BCO-associated EC isolates, with SS isolates favoring genomic plasticity and rapid systemic dissemination, while BCO isolates are metabolically specialized for chronic persistence and survival in the bone environment.

## 5 Conclusion

The discriminatory k-mers identified in this study should be interpreted as statistical signatures associated with disease-linked isolates rather than fixed determinants of pathogenicity, particularly given the known genomic plasticity of *Enterococcus* species (48). In our analysis, the extended regions surrounding many identified variant loci appeared relatively conserved (comparison of ten amino acids before and after k-mers of interest, data not shown). This observation suggests that these sites may occur within stable sequence contexts, although it was qualitative and not explored further in the present study. However, not all k-mers were derived from such regions; some reflect allelic variation within orthologous genes, while others are associated with the presence or absence of accessory genes or with orthologs that are unevenly distributed across isolates. Together, these patterns suggest that both localized amino acid variation and broader gene content differences may contribute to phenotypic differences across EC pathotypes. Importantly, these findings should not be interpreted as defining strict boundaries between commensal and pathogenic EC isolates, but rather as identifying proteomic signatures associated with disease-linked isolates. The *cpsO*-based assays that test a biologically defined capsular-locus marker were recently used to distinguish commensal from pathogenic isolates (49, 50). Our approach differs in that it was not designed around a preselected pathogenic locus; instead, the candidate markers were obtained from a data-driven joint-prevalence analysis of protein k-mers across the pathogenic and commensal groups. The absence of *cpsO*-derived k-mers among our selected features does not contradict the biological relevance of *cpsO*; rather, it implies that other proteomic signatures provided stronger discrimination within this dataset.

While discriminatory k-mers were consistently identified between commensal and pathogenic isolates with known functions, some k-mers (DSGTI and RKEKI) mapped to proteins annotated only as hypothetical across multiple annotation resources (NCBI BLASTP, UniProt, Bakta, InterProScan, and AlphaFold), reflecting gaps in current functional annotation. This limits the immediate mechanistic interpretation of their role in pathogenesis. In addition, since the analysis was restricted to proteome-derived sequences, it does not capture the contribution of non-coding regulatory elements. Future studies should therefore focus on functional validation of candidate proteins, particularly those annotated as hypothetical, through experimental assays. Expanding isolate collections across production systems and geographies will improve model generalizability, while integration with multi-omics data will help link sequence features to functional traits. Such efforts will refine the causal understanding of pathogenicity and support the development of diagnostic tools for rapid surveillance of EC pathotypes. Another limitation of this study is the reliance on publicly available genome assemblies and associated metadata, which may contain inconsistencies in disease labeling. Although metadata were manually curated to improve consistency, misclassification cannot be fully excluded. It is important to conduct future studies using well-characterized isolates from both healthy and diseased birds to strengthen confidence in the biological and diagnostic relevance of these proteomic signatures.

Our comparative proteomic analysis across commensal, SS-associated, and BCO-associated EC isolates reveals proteomic signatures underlying divergent pathogenic behaviors. Compared with commensal isolates, both SS and BCO isolates show enrichment for proteins involved in mobile genetic elements and metabolic adaptation, reflecting a shift toward host-associated pathogenic lifestyles. SS isolates are further distinguished by proteins associated with DNA methylation and the potential for rapid systemic invasion, traits that likely facilitate acute disease in young broilers. In contrast, BCO isolates display enrichment for carbohydrate transporters, metabolic enzymes, and tissue-persistence factors, consistent with chronic colonization of bone tissue in older birds. These findings show that pathogenic EC isolates are not uniform but instead exhibit distinct, niche-specific proteomic adaptations, offering critical insights into their evolution, epidemiology, and potential control strategies. Supervised learning approaches have proven instrumental in resolving these pathotypes, highlighting their utility in the surveillance and management of emerging poultry pathogens.

## Data Availability

The original contributions presented in the study are included in the article/Supplementary material, further inquiries can be directed to the corresponding author.
